# Utility of dual‐chamber Electrogram‐based pace mapping in a teenager with a focal atrial tachycardia, low inducibility, and indeterminate earliest excitation site

**DOI:** 10.1002/joa3.13197

**Published:** 2024-12-05

**Authors:** Mitsuhiko Shoda, Mitsuru Takami, Kimitake Imamura, Koji Fukuzawa

**Affiliations:** ^1^ Division of Cardiovascular Medicine Department of Internal Medicine Kobe University Graduate School of Medicine Kobe Japan; ^2^ Section of Arrhythmia, Division of Cardiovascular Medicine Department of Internal Medicine Kobe University Graduate School of Medicine Kobe Japan

**Keywords:** catheter ablation, focal atrial tachycardia, pace mapping

## Abstract

A 17‐year‐old patient presented with frequent palpitations, where the tachycardia was not sustained and could not be induced, making it impossible to pinpoint the earliest activation site using the activation map. However, by utilizing a dual‐chamber electrogram‐based pace mapping technique, we successfully identified the origin and achieved effective treatment.

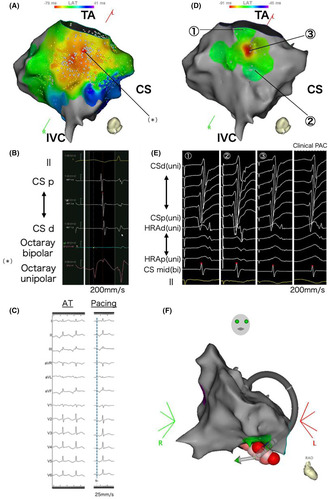

The patient, a 17‐year‐old female, had been experiencing recurrent palpitations for several months and was diagnosed with a long RP' narrow QRS tachycardia. She had no structural heart disease. The P′ wave was negative in the inferior leads and exhibited a saw‐tooth morphology. The chest leads showed negative concordance on the 12‐lead electrocardiogram (ECG) (Figure 1). As medical treatment did not reduce the frequency of tachycardia, catheter ablation was performed. Despite frequent palpitations before the procedure, no tachycardia was observed or induced during the session. The electrophysiological study revealed no ventriculoatrial conduction. Premature atrial contractions (PACs) with the same morphology as the P′ wave observed in the preoperative ECG were detected, leading us to infer that this tachycardia was a focal atrial tachycardia (FAT). Activation mapping of those PACs was performed, but their frequency was too low to identify the earliest activation site (EAS). Several radiofrequency (RF) applications were delivered from the coronary sinus (CS) ostium to the inferior vena cava, targeting the earliest activation area within the evaluable range, before concluding the session. However, the tachycardia recurred within days, and daily palpitations significantly affected her school life, leading to a second catheter ablation.

**FIGURE 1:**
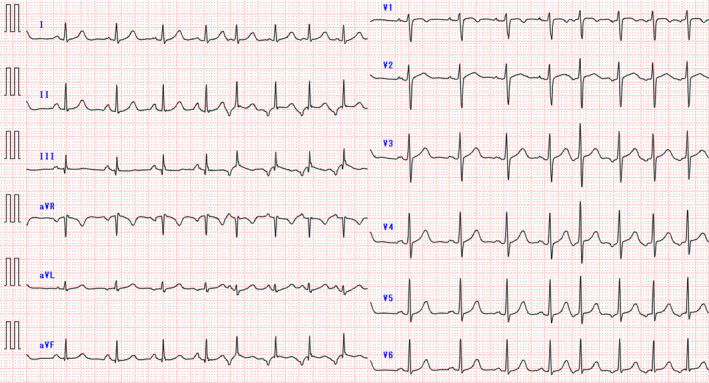
This 12‐lead electrocardiogram exhibits a long RP' narrow QRS tachycardia. The P′ wave was negative in the inferior leads and exhibited a saw‐tooth morphology. The chest leads exhibited negative concordance of the P′ wave.

The second catheter ablation was performed using an open‐irrigated contact force‐sensing catheter (QDOT Micro™ catheter; Biosense Webster, CA, USA) and multisplines mapping catheter (Octaray; Biosense Webster) with an electroanatomical mapping system (CARTO 3, Biosense Webster). A 6F 20‐pole electrode catheter (BeeAT; Japan Lifeline, Tokyo, Japan) was inserted through the right jugular vein into the CS as a reference to obtain dual‐chamber electrograms from the CS and right atrium (RA). Although she experienced frequent palpitations before the procedure, no atrial tachycardia was observed once the EPS began. Figure 2A illustrates an activation map of the low RA created with a limited number of PACs, as an insufficient number of PACs was available. The EAS was not clearly identifiable and the unipolar electrogram at the EAS within the mapped area showed an rS pattern, not a QS pattern (Figure 2B). As a result of the insufficient number of mapped PACs, the QS pattern was not observed within the mapped area. During this mapping mechanical stimulation from the mapping catheter eliminated the infrequent PACs, resulting in a blurred activation map and making it challenging to further pinpoint the EAS. Therefore, we searched for the origin of the FAT using a pace mapping technique known as the Intracardiac Pace Match Scoring (iPASO) technique, using dual chamber unipolar electrograms as a reference with the Intracardiac Pattern‐Matching (ICPM) software (CARTO 3; Biosense Webster), which multiplies the matching score by (−100 ms) and reflects it on the local activation time (LAT) map. Focusing on the area of interest, we performed pace mapping using minimal output to capture solely the local myocardium. The highest ICPM matching score of 0.91 was found on the cavotricuspid isthmus (CTI), 10.3 mm away from the annulus at the 6 o'clock position of the tricuspid valve. The P wave during pacing at the highest‐scoring site on the 12‐lead ECG had the same polarity as the P wave during tachycardia in all leads. Detailed pace mapping was performed around the highest‐scoring site, where the scores for the surrounding areas were significantly lower, showing a centrifugal pattern on the iPASO map (Figure 2C,D). Finally, pace mapping was performed at 17 points, with matching scores ranging from 0.68 (−68 ms) to 0.91 (−91 ms). RF applications were delivered around the center of the iPASO map, followed by linear ablation along the CTI. Bidirectional block was confirmed to prevent the occurrence of CTI‐dependent macro‐reentry AT because of iatrogenic conduction delays from the ablation. Isoproterenol was administered, and atrial burst pacing was repeated; however, no atrial tachyarrhythmias were observed. The patient remained free from any tachyarrhythmias for 6 months after the procedure.

**FIGURE 2:**
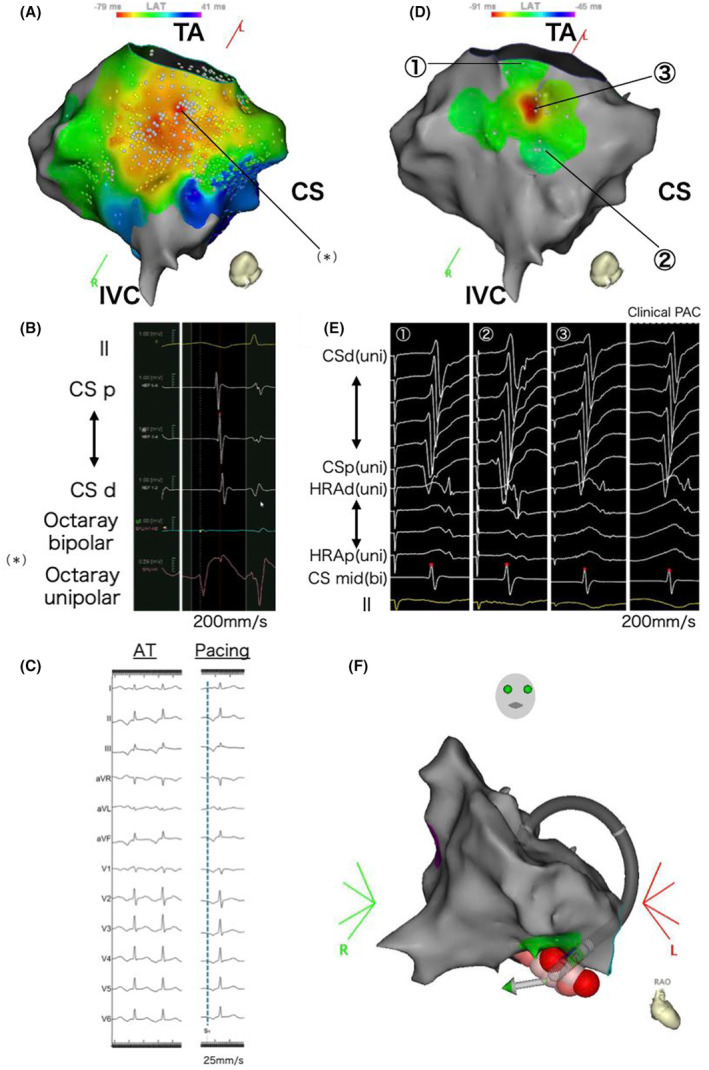
(A) The activation map (LAO caudal) shows early activation in the lower RA, but the exact earliest activation site could not be identified. The (*) indicates the earliest activation sites within the mapped area. (B) In the electrogram from the earliest activation site (*) recorded by the Octaray mapping catheter, unipolar potentials presented an rS pattern, with no QS pattern detected. (C) The left panel shows the P wave during tachycardia, and the right panel shows the P wave during pacing at the highest‐scoring site. The polarity matched across all leads. (D) iPASO map (LAO caudal). The highest ICPM matching score is found on the CTI, 10.3 mm away from the tricuspid valve. Further, the scores for the surrounding areas are lower, with the iPASO map exhibiting a centrifugal pattern. (E) The dual‐chamber (RA and CS) unipolar electrograms obtained from pace mapping at the three sites shown in Figure 2C, reference EGMs of the clinical PAC, and matching scores calculated by the ICPM. The matching scores for the three sites calculated by the ICPM were ① 0.75, ② 0.70, and ③ 0.91, respectively. (F) RF application sites on the three‐dimensional anatomical map (RAO view). During RF application, the ablation catheter was positioned deeper than the geometry, suggesting a pouch structure in the CTI. RA = Right Atrium; CS = Coronary Sinus; CTI = Cavotricuspid Isthmus; ICPM = Intracardiac Pattern‐Matching; iPASO = Intracardiac Pace Match Scoring; LAO = Left Anterior Oblique; RAO = Right Anterior Oblique; PAC = Premature Atrial Contraction.

Originally, Yamashita et al.^1^ reported the technique of iPASO mapping to detect nonpulmonary vein trigger PACs. In this teenager's case, the iPASO mapping technique may also allow for effective ablation with fewer applications in the FAT ablation procedure, where identifying the earliest excitation site is challenging. One of the most important aspects of a successful ablation of FAT is to localize the EAS. However, when a FAT is difficult to induce and nonsustained, the treatment becomes clinically challenging. Extensive ablation may lead to the formation of arrhythmogenic substrates and risk injury to the conduction system. In young patients, minimizing the number of ablation applications is particularly desirable. For identifying FAT origin with very low inducibility, traditional atrial pace mapping techniques face limitations because of the small and dull P wave potentials compared to the QRS morphology. Man et al.^2^ reported that the spatial resolution of atrial pace mapping is approximately 17 mm, which is clinically inadequate. Hayashi et al.^3^ reported that combining the P‐wave and bipolar electrogram sequences from the His region and CS enables a more detailed pace mapping. However, that requires a manual analysis, as it is not automated. The iPASO technique overcomes those limitations, allowing for a more precise pace mapping. The iPASO map using dual‐chamber electrograms as a reference that reflects the matching score calculated by the ICPM software without operator intervention on the LAT map allows for detailed pace mapping.

In this case, the FAT originated from the CTI, an uncommon site for FATs. Sato et al.^4^ reported that the CTI accounts for approximately 3% of all FATs and that a CTI‐origin FAT has unique electrocardiographic characteristics such as saw‐tooth morphology, as seen in our case. Further, a CTI pouch is a common anatomical anomaly found in 16% of autopsied human hearts^5^. In our case, the complex anatomical structure of the CTI, as a result of the pouch, made creating an activation map and identifying the FAT origin challenging. As shown by the ablation catheter positioned deeper than the geometry on the three‐dimensional anatomical map (Figure 2E), there was a pouch structure in the CTI, indicating insufficient contact of the mapping catheter for an accurate activation map. Additionally, mechanical stimulation by the mapping catheter occasionally suppressed the tachycardia, further complicating the process. The PACs had already disappeared before ablation, making it difficult to precisely point out the effective ablation site. However, in this case, where extensive EAS were identified on the activation map, the iPASO map was extremely useful in determining the ablation point.

## CONCLUSION

In situations where traditional activation mapping cannot pinpoint the earliest excitation site of tachycardia, the iPASO mapping may effectively identify its origin more precisely.

## FUNDING INFORMATION

This research did not receive any specific grant from funding agencies in the public, commercial, or not‐for‐profit sectors.

## CONFLICT OF INTEREST STATEMENT

The Section of Arrhythmia (from the Division of Cardiovascular Medicine, Department of Internal Medicine, Kobe University Graduate School of Medicine, Kobe, Japan) is supported by an endowment from Medtronic JAPAN, Abbott JAPAN, and Boston Scientific JAPAN. K.I. and K.F. belong to the Section. However, all authors have reported that they have no relationships relevant to the contents of this paper to disclose.

## ETHICS STATEMENT

Not applicable.

## PATIENT CONSENT STATEMENT

Patient gave consent to publish this case report.

## CLINICAL TRIAL REGISTRATION

Not applicable.

## Data Availability

The data that support the findings of this study are available from the corresponding author upon reasonable request.
